# Beyond Racial Categorization in Sports Cardiology: A Systematic Review of Cardiac Adaptations in Athletes

**DOI:** 10.3390/jcm14197107

**Published:** 2025-10-09

**Authors:** Douglas Corsi, Rafael Hernandez, Jasmine Yimeng Bao, Stephen Garrova, David Shipon

**Affiliations:** 1 Rutgers Robert Wood Johnson Medical School, New Brunswick, NJ 08901, USA; 2 Sidney Kimmel Medical College, Thomas Jefferson University, Philadelphia, PA 19107, USA; rafael.hernandez@students.jefferson.edu (R.H.); yimeng.bao@students.jefferson.edu (J.Y.B.); stephen.garrova@students.jefferson.edu (S.G.); 3 Department of Cardiology, Thomas Jefferson University Hospital, Philadelphia, PA 19107, USA; david.shipon@jefferson.edu

**Keywords:** sports cardiology, athlete’s heart, racial disparities, electrocardiography, echocardiography, sudden cardiac death, health equity, cardiac screening

## Abstract

**Background/Objectives:** Race-based cardiac screening criteria in sports cardiology, including the “Black athlete’s heart” concept, assume biological distinctions that may not reflect physiological reality. This systematic review evaluates whether geographic ancestry provides more clinically relevant predictors of cardiac adaptation than racial categorization. **Methods:** PubMed was searched (January 2005–July 2025) for studies examining cardiac adaptations in athletes by ethnicity. Data extraction captured demographics, geographic origin, cardiac assessments, and outcomes. Narrative synthesis was employed due to methodological heterogeneity. **Results:** Forty-seven studies (*n* = 66,130) revealed substantial within-race heterogeneity. The “Black athlete repolarization variant” prevalence ranged from 1.8% (Brazilian) to 30% (Ghanaian) Black athletes. Left ventricular wall thickness >12 mm (normal <11 mm) occurred in 7.1% of Black versus 0.4% of White athletes, yet varied significantly within Black populations—10.8 ± 1.2 mm in Sub-Saharan versus 9.4 ± 1.1 mm in African-American athletes (*p* < 0.001). Relative wall thickness ≥0.44 (normal ≤0.42) was presented in 43% of West/Middle African, 23% of East African, and 7% of White athletes. T-wave inversion showed four-fold variation within Black populations (3.6–8.5% West African versus 0.5–2.0% African-American/Caribbean). Current International Criteria demonstrated inequitable specificity: 3.3% false-positive rate in Black versus 1.4% in White athletes. **Conclusions:** Geographic ancestry explains more cardiac variation than racial categories, supporting contemporary understanding of race as a sociopolitical construct. The persistent diagnostic disparities in ECG screening specificity highlight the need for reform. Transitioning toward protocols incorporating continental origin, anthropometric factors, and social determinants of health—while eliminating terminology like “Black athlete’s heart”—represents an important step toward achieving equity in cardiovascular care for diverse athletic populations.

## 1. Introduction

The concept of the “athlete’s heart” represents a well-established constellation of physiological cardiac adaptations resulting from intensive athletic training. However, the recognition of ethnicity-specific electrocardiographic and echocardiographic patterns has led to the controversial concept of the “Black athlete’s heart,” which assumes distinct cardiac adaptations in athletes of African descent. This terminology perpetuates problematic racial essentialism in medicine and implies that physiological variations represent pathological deviations from a “normal” (implicitly White) standard. Contemporary sports cardiology must move beyond such race-based nomenclature, which lacks scientific precision and may contribute to health disparities. This systematic review critically examines the evidence supporting race-based cardiac screening criteria in athletes, evaluating both electrocardiographic and echocardiographic findings attributed to Black athletes while questioning the scientific validity and clinical implications of race-based medical algorithms in sports cardiology. Additionally, this systematic review examines whether geographic ancestry provides more clinically relevant predictors of cardiac adaptation than broad racial categories. We hypothesize that athletes from different continental regions will demonstrate distinct patterns of ECG repolarization variants and left ventricular hypertrophy, and that current race-based screening criteria perpetuate diagnostic disparities. The primary purpose is to evaluate the scientific validity of racial categorization in sports cardiology and provide evidence for more equitable screening protocols. Throughout this review, references to “Black athletes” or similar racial terminology reflect the language used in the existing literature being analyzed and should not be interpreted as an endorsement of such categorization for future clinical practice or research.

## 2. Materials and Methods

This systematic review was conducted according to PRISMA 2020 guidelines, as detailed in the Supplementary Materials. This review was not prospectively registered as it was designed as an exploratory analysis of existing literature patterns rather than a hypothesis-testing study. A detailed study protocol was not prepared for this review.

### 2.1. Search Strategy and Study Selection

We conducted a comprehensive literature search of the PubMed database from 1 January 2005, to 31 July 2025. The search strategy employed combinations of MeSH terms and keywords including: (“Black” OR “African” OR “Afro-Caribbean” OR “African American”) AND (“athlete*” OR “sport*” OR “exercise”) AND (“electrocardiograph*” OR “ECG” OR “echocardiograph*” OR “cardiac adaptation*” OR “athlete’s heart”).

### 2.2. Data Synthesis

Due to substantial heterogeneity in study populations (geographic origin, sport types), assessment methods (varying ECG criteria), and outcome reporting, formal meta-analysis was deemed inappropriate. Data synthesis followed a narrative approach due to heterogeneity in study populations, outcome measures, and statistical reporting. Where possible, prevalence rates and mean differences with confidence intervals were extracted for comparative analysis.

### 2.3. Assessment of Study Quality and Evidence Certainty

Given the narrative focus of this review and the substantial heterogeneity in study designs, populations, and outcome measures across the included observational studies, formal risk of bias assessment using standardized tools (e.g., Newcastle-Ottawa Scale) and certainty of evidence evaluation (e.g., GRADE) were not conducted. The primary objective was to critically examine existing literature patterns and concepts rather than to synthesize evidence for specific clinical recommendations. Study limitations and methodological considerations are discussed qualitatively within the results and limitations sections.

## 3. Results

**Figure 1:**
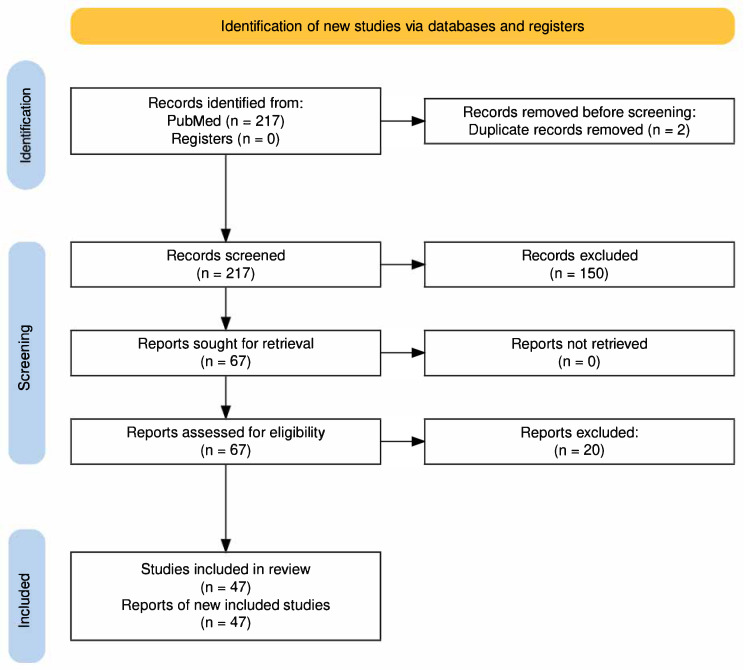
PRISMA Flow Diagram for Study Selection.

The literature search identified, 43 primary studies and 4 systematic reviews met inclusion criteria, encompassing 66,130 total participants across individual studies (Table 1, Figure 1). Two independent reviewers extracted data using standardized forms capturing: study design, athlete demographics (including geographic origin when specified), sport type, training level, cardiac assessment methods, and primary outcomes. Discrepancies were resolved through consensus with a third reviewer.

**Table 1:** Summary of Included Studies.

Study (Author & Number)	Year	Design	Population N	(Black)	Sport(s)	Geographic Origin	Population Key	Comparator(s)/Purpose
ECG Studies								
Sokunbi et al. [1]	2021	Cross-sectional observational comparative study	360	360	Multiple	Nigeria	Adolescent athletes and controls	Prevalence/distribution of ECG patterns; athletes vs. controls; training vs. non-training findings
Sheikh et al. [2]	2014	Retrospective cross-sectional diagnostic accuracy	5505	1208	Mixed	UK	Black/White athletes, HCM	ESC vs. Seattle vs. refined ECG criteria
Conway et al. [3]	2022	Retrospective cross-sectional diagnostic accuracy	1686	123	Mixed	USA	NCAA Division I athletes	Seattle vs. Refined vs. International ECG criteria
Lander et al. [4]	2024	Cross-sectional observational	173	129	Basketball	USA	Elite female professional basketball athletes (WNBA)	Reference ECG range/prevalence of findings
Malhotra et al. [5]	2020	Retrospective cross-sectional diagnostic accuracy	11,168	1005	Soccer	UK	Adolescent soccer players (white/black)	ESC vs. Seattle vs. refined vs. international ECG criteria
McClean et al. [6]	2019	Retrospective cross-sectional diagnostic accuracy	1304	428	Mixed	UK/Qatar	Arab/black male pediatric athletes (11–18 years)	2010 ESC vs. Seattle vs. international ECG criteria
Zorzi et al. [7]	2022	Cross-sectional observational comparative study	2229	1115	Multiple	Italy	Young competitive athletes	Prevalence/clinical significance of isolated low QRS voltage
Miragoli et al. [8]	2019	Retrospective cross-sectional observational	414	69	Mixed	Italy	Non-professional adolescent athletes	Prevalence/correlates of early repolarization pattern
Junttila et al. [9]	2011	Cross-sectional observational study	503	151	Multiple	USA	Young collegiate athletes	Prevalence/characteristics of inferolateral early repolarization; association with LVH, gender
Papadakis et al. [10]	2011	Prospective longitudinal observational cohort	2894	904	Mixed	UK	Black/White athletes, controls, HCM patients	ECG patterns/incidence of HCM by group
Ferrari et al. [11]	2024	Multicentre retrospective cross-sectional observational	6125	1625	Soccer	Brazil	Male Brazilian football players	Prevalence/correlates of abnormal ECG/imaging findings
Pambo et al. [12]	2021	Cross-sectional observational	159	159	Soccer	Ghana	Male competitive athletes	Cardiac findings by geographic/ethnic subgroup
Pambo et al. [13]	2021	Cross-sectional observational	75	75	Soccer	Ghana	Female competitive athletes	Cardiac findings by geographic/ethnic subgroup
Riding et al. [14]	2019	Cross-sectional observational comparative	1698	1019	Mixed	Multi-regional	Adolescent athletes and healthy controls	Prevalence and significance of T-wave inversions
Muramoto et al. [15]	2014	Retrospective cross-sectional observational comparative	1114	71	Multiple	USA	Varsity athletes	Prevalence, pattern, and prognostic significance of J-wave/early repolarization by group, race, and sex
Papadakis et al. [16]	2009	Cross-sectional observational comparative	2110	65	Mixed	UK	Healthy athletes, HCM patients, ARVC patients	ECG repolarization markers for distinguishing physiological vs. pathological anterior T-wave inversion
Calore et al. [17]	2016	Cross-sectional observational comparative	233	53	Mixed	Italy	Pre-adolescent athletes undergoing ECG screening	Prevalence and natural history of T-wave inversion subtypes
D’Ascenzi et al. [18]	2019	Prospective longitudinal observational cohort	2227	0	Mixed	Italy	Healthy athletes vs. ARVC patients, matched for age, sex, ethnicity	ECG markers for distinguishing athlete’s heart from ARVC
McClean et al. [6]	2019	Cross-sectional comparative diagnostic accuracy	732	314	Mixed	UK/Qatar	Arab and black male pediatric athletes (11–18 years)	Diagnostic accuracy: international vs. refined ECG recommendations for ATWI
Brosnan et al. [19]	2018	Matched cross-sectional comparative observational	200	3	Mixed	Multi-national	Mixed-race, Black, and White adolescent male soccer players	Cardiac electrical/structural adaptation by race/ethnicity
Jacob et al. [20]	2015	Prospective cross-sectional observational study	1755	352	Multiple	USA	Collegiate athletes	Prevalence and significance of isolated T wave inversion
Malhotra et al. [21]	2021	Cross-sectional observational comparative	3000	1000	Soccer	UK	Elite male American football players (NFL Combine)	ECG abnormalities by race and player position
Wilson et al. [22]	2012	Cross-sectional observational comparative study	1220	300	Multiple	Qatar/West Asia	National-level male athletes	ECG abnormalities
Bryde et al. [23]	2025	Cross-sectional observational comparative study	706	85	Soccer	USA	MLS professional athletes	ECG findings by ethnicity; targeted echo follow-up for abnormalities
Magalski et al. [24]	2008	Cross-sectional observational comparative	1959	1321	American Football	USA	Competitive collegiate athletes	Incremental value of ECG and echocardiography for preparticipation screening
Magalski et al. [25]	2011	Prospective cross-sectional observational	964	188	Mixed	USA	Professional male athletes (various sports, ethnicities)	ECG findings by sport and ethnicity
Raman & Vyselaar [26]	2022	Cross-sectional observational comparative	753	285	Mixed	Canada	Male African American basketball players/youth athletes	Cardiac screening findings by athlete group
Crouse et al. [27]	2009	Cross-sectional observational comparative study	77	54	American football	USA	NCAA Division I football athletes	Prevalence and types of ECG abnormalities by race
Grace et al. [28]	2015	Cross-sectional observational comparative study	45	45	Boxing, Body building	South Africa	University students of Zulu descent	ECG patterns: endurance vs. resistance vs. controls; prevalence of LVH & repolarization changes
Rambarat et al. [29]	2020	Multicenter retrospective cohort study	329	64	NCAA Division I (multiple)	USA	Collegiate female athletes	Preparticipation cardiac screening: sport, race differences in ECG/echo parameters
Corsi et al. [30]	2025	Retrospective cross-sectional observational comparative	8303	200	Basketball	USA	American collegiate football players	Cardiac remodeling: pre- vs. post-training echocardiography
Echocardiographic Studies								
Hamburger et al. [31]	2023	Prospective longitudinal observational cohort	85	52	American Football	USA	Collegiate athletes	Utility of echocardiography as primary screening
Engel et al. [32]	2016	Cross-sectional observational study	526	406	Basketball	USA	NBA players	Cardiac structure and function by race/anthropometry
Basavarajaiah et al. [33]	2008	Cross-sectional observational comparative	900	450	Mixed	UK	Black athletes (by region), comparator non-Black	Cardiac electrical/structural patterns by geographic/ethnic origin
Gjerdalen et al. [34]	2014	Cross-sectional observational comparative study	553	49	Soccer	Norway/Scandinavia	Male professional football players	Cardiac chamber remodeling by ethnicity; BSA-indexed LV/RV measurements
Pelà et al. [35]	2015	Cross-sectional observational comparative	138	41	Soccer	Italy	Amateur footballers (West-African Black/Italian White)	LV structural remodeling by ethnicity
Tso et al. [36]	2022	Prospective longitudinal observational cohort	249	124	American Football	USA	Collegiate football athletes (Black/White)	Association of race and position with acquired concentric LVH over time
Moneghetti et al. [37]	2019	Cross-sectional observational comparative study	230	98	American football	USA	NCAA Division I ASF players	Race differences in LV remodeling (mass-to-volume, sphericity, strain, etc.)
Di Paolo et al. [38]	2012	Cross-sectional observational comparative	216	154	Mixed	Sub-Saharan Africa	Adolescent African/Italian soccer players	ECG and echocardiographic findings by ethnicity and country
Crouse et al. [39]	2016	Cross-sectional observational comparative	80	36	American Football	USA	Collegiate ASF athletes	Echo/BP characteristics vs. reference and by ethnicity
Dzudie et al. [40]	2007	Cross-sectional observational comparative	21	21	Handball	Cameroon	Elite handball players/controls	Cardiac structure/function by athletic status
Kervio et al. [41]	2013	Cross-sectional observational comparative	282	96	Soccer	Multi-national	Japanese, African-Caribbean, Caucasian soccer players	ECG/echo characteristics by ethnicity
Cho et al. [42]	2019	Cross-sectional observational comparative study	1185	140	Multiple	International (South Korea)	University athletes (Universiade)	Incidence and predictors of abnormal LV geometry by race, sport type, and training time
Edenfield et al. [43]	2019	Retrospective cross-sectional observational comparative	375	218	American Football	USA	Collegiate football players	ARD by position, race, BSA; generation of BSA-specific ARD norms
Augustine et al. [44]	2024	Cross-sectional observational comparative	1087	163	Soccer	UK	Adolescent academy footballers (White/Black/Mixed-race)	RV dimension and ECG features by ethnicity; prevalence/overlap with ARVC criteria
Zaidi et al. [45]	2013	Cross-sectional observational comparative	675	297	Mixed	UK	Black/White athletes, sedentary controls	RV structure/function and ECG findings by ethnicity and activity
ECG + Electrocardiogram Studies								
Waase et al. [46]	2018	Coress-sectional observational comparative study	519	409	Basketball	USA	NBA athletes	Generate normative ECG data for elite professional athletes/Assess athlete ECG interpretation criteria
Sheikh et al. [47]	2013	Cross-sectional observational comparative study	329	329	Multiple	UK	Adolescent Black athletes	LVH and ECG repolarization changes
Schmied et al. [48]	2009	Cross-sectional observational screening study	155	155	Soccer	Algeria	U-17 football players	Precompetition cardiac screening: prevalence & ethnic variation in ECG/echo findings; risk factors for SCD
Uberoi et al. [49]	2013	Cross-sectional observational comparative study	85	Not specified	American football	USA	NCAA football players	Cardiac dimensions and ECG/echo remodeling by lineup position and race
Haddad et al. [50]	2013	Cross-sectional observational comparative study	112	38	American football	USA	NCAA Division I football players	Race differences in ventricular mass/volume ratio, function, and ECG
Demola et al. [51]	2019	Cross-sectional observational comparative study	90	30	Multiple	Italy	Early adolescent athletes	Ethnicity-related differences in hemodynamic and ECG adaptation to exercise; relation to LV remodeling
Di Gioia et al. [52]	2024	Prospective longitudinal observational cohort study (cross-sectional & comparative analyses)	1492	57	Multiple sports (including endurance)	Italy	Olympic elite athletes	Prevalence, morphology, and prognosis of LVTs; comparisons by sex, race/ethnicity, and sport type
Reviews								
Davis et al. [53]	2022	Systematic Review	51 studies; 65,629 individuals	Variable	Mixed	Multi-national	Athletes	Ethnic differences in athlete ECGs, focus on T-wave inversion and race/ancestry impact on ECG interpretation
McClean et al. [54]	2018	Meta-analysis	43 studies; 16,396 individuals	Variable	Mixed	Multi-national	Pediatric Athletes	Impact of age, race, and sex on electrical and structural cardiac remodeling in pediatric athletes
Pambo & Scharhag [55]	2021	Systematic Review	16 studies; 5632 individuals	Variable	Mixed	Multi-national	Black African and Afro-Caribbean athletes	ECG/ECHO findings in Black athletes; prevalence and characteristics of repolarization and hypertrophy patterns
Christou et al. [56]	2020	Systematic Review	58 studies; 7221 individuals	Variable	Multiple	Multi-national	Athletes	Impact of demographic, anthropometric, and athletic factors on left atrial size in athletes

The included studies represented diverse geographic regions and athletic populations. Geographic distribution included studies from the United States (*n* = 14), United Kingdom (*n* = 9), Italy (*n* = 6), Ghana (*n* = 2), multi-national collaborations (*n* = 5), Brazil (*n* = 1), Norway (*n* = 1), Cameroon (*n* = 1), Qatar (*n* = 1), Algeria (*n* = 1), South Africa (*n* = 1), and South Korea (*n* = 1). Study designs comprised cross-sectional observational studies (*n* = 29), retrospective cross-sectional studies (*n* = 8), prospective longitudinal observational cohort studies (*n* = 4), multicenter retrospective cohort studies (*n* = 1), matched cross-sectional comparative observational studies (*n* = 1), and systematic reviews (*n* = 4).

### 3.1. Study Populations and Participant Characteristics

The total study population included approximately 25,500 Black athletes across all primary studies, with individual study sizes ranging from 21 to 11,168 participants. The largest single study contributed 11,168 participants (10,163 White, 1005 Black soccer players), while the smallest focused studies included populations of 21–75 Black athletes.

Sport distribution encompassed soccer/football (*n* = 18 studies), American football (*n* = 9 studies), basketball (*n* = 4 studies), mixed sports (*n* = 11 studies), and handball (*n* = 1 study). The majority of studies (*n* = 37) focused on male athletes, with limited representation of female athletes (*n* = 3 studies) and mixed-sex cohorts (*n* = 3 studies).

Geographic ancestry of Black athletes was specified in 21 studies, including West African (Ghana, *n* = 2; Nigeria, *n* = 1; Algeria, *n* = 1), Middle African (Cameroon, *n* = 1), Sub-Saharan African (*n* = 1), South African (*n* = 1), African-American/Caribbean (*n* = 12), and mixed African heritage populations (*n* = 2). Twenty-two studies did not specify geographic ancestry beyond broad racial categorization.

### 3.2. Electrocardiogram Findings in Black Athletes

#### The Black Athlete Repolarization Variant

ER is a normal finding defined by the IC as elevation of the QRS-ST junction (J point) by ≥0.1 mV with late QRS slurring or notching (J wave) in inferolateral leads [57]. Spatial heterogeneity in the dispersion of myocardial refractory periods is thought to underlie ER, driven by a net outward repolarizing current [58]. In athletes, physiological adaptations such as increased vagal tone, shown to enhance the epicardial action potential notch, and concentric left ventricular (LV) remodeling (including LVH voltage criteria) have been associated with increased prevalence of ER [8,9,48,59,60,61,62]. The “Black athlete repolarization variant” represents a specific combination of ECG findings due to early repolarization, determined to be a normal finding in Black athletes [10,59]. This pattern has been endorsed by the Seattle, refined, and IC and remains the only ethnicity-based criterion in current guidelines [2,53,57,60,61]. Multiple studies confirmed the prevalence of this repolarization variant in Black athletes, though prevalence data reveal substantial geographic heterogeneity that challenges the utility of broad racial categorization (Table 2) [4,11,12,13,14,15,63]. While studies in Brazil and the U.S., involving athletes with potentially more geographically diverse backgrounds, have reported prevalence of 1.8% among Black male soccer players and 9.3% among Black female basketball players, higher prevalence has been observed in athletes from Nigeria (29.3%) and Ghana (30% in males, 18.3% in females), as well as in broader regional cohorts from Middle Africa (1.8%) and West Africa (5.3%) [4,11,12,13,14,63]. This geographic variation suggests that continental ancestry may be more relevant than self-identified race in determining ECG patterns.

**Table 2:** Summary of differential ECG findings in Black and non-Black athletes.

ECG Finding	IC Classification	In Black Athletes	In Athletes of Other Ethnicities
Sinus bradycardia	Normal	Mixed findings in prevalence vs. White athletes [5,10,21]	Mixed findings in White athletes vs. Black athletes; more prevalent in mixed-race athletes than both Black and White athletes [5,10,21]
Incomplete RBBB	Normal	Increased prevalence vs. White athletes [10]	Less prevalent in White athletes [10]
Complete RBBB	Borderline	Decreased prevalence vs. White athletes [10]	More prevalent in White athletes [10]
Voltage criteria for LVH or RVH	Normal	LVH: less prevalent in soccer players; more prevalent in football players and athletes of African-American/Caribbean, Middle African, and West African descent [5,14,21,24,25,26]; RVH: increased prevalence vs. White athletes, more pronounced in Middle Africans [5,10,14,21]	LVH: more prevalent in White and mixed-race soccer players; less prevalent in White football players [5,21,24,25,26]; RVH: less prevalent in White athletes, comparable in mixed-race athletes [5,10,21]
Voltage criteria for LAE or RAE	Borderline	Increased prevalence of left atrial enlargement (LAE) and right atrial enlargement (RAE) vs. White athletes [5,10,21,22]	Less prevalent in White athletes; comparable in mixed-race athletes vs. Black athletes [5,21]
Right axis deviation	Borderline	Decreased prevalence vs. White athletes [10]	More prevalent in White athletes [10]
1º AV block	Normal	Increased prevalence vs. White athletes [5,10]	Less prevalent in White athletes [5]
ER/STE	Normal	Black athlete repolarization variant: convex STE followed by TWI in V1-V4, more prevalent in Middle Africans [4,10,14,57,59,61]; Increased prevalence of ER vs. White athletes [8,15,26]; Increased prevalence of STE, including ascending convex and ascending concave, vs. White athletes [10,21,49]; Increased prevalence of nonspecific ST changes vs. athletes of other ethnicities [26,30]	Decreased prevalence of ER in White athletes [8,26]; Decreased prevalence of all STE in White and mixed-race athletes; comparable ascending concave STE in Black and mixed-race athletes [10,21]; Decreased prevalence of nonspecific ST changes in non-Black athletes [26,30]
TWI	Abnormal (except Black athlete repolarization variant and juvenile T-wave pattern)	Black athlete repolarization variant: benign TWI following convex STE in V1-V4, more pronounced in Middle and West Africans [2,4,10,11,14,49,59,61,64]; Increased prevalence of abnormal TWI in inferior/lateral leads vs. White and/or mixed-race athletes; most pronounced in Middle and West Africans [5,10,11,14,21,22,24,30]	Less prevalent in non-Black athletes, including those identifying as White and mixed-race [5,10,11,21,30]
ST segment depression	Abnormal	Increased prevalence vs. non-Black athletes [10,30]	Less prevalent in non-Black athletes [10,30]

### 3.3. T-Wave Inversion Patterns

The IC define abnormal TWI as ≥1 mm in depth across at least two contiguous leads [57]. Abnormal TWI in inferolateral leads is linked to hypertrophic cardiomyopathy (HCM), while TWI in leads V1-V3 is associated with arrhythmogenic right ventricular cardiomyopathy (ARVC) [57]. These patterns reflect repolarization abnormalities due to hypertrophy-related action potential prolongation and ionic remodeling in HCM, or fibrofatty myocardial replacement and right ventricular (RV) dilatation in ARVC [57,62,64]. Although TWI is generally considered a training-unrelated abnormal finding warranting clinical investigation, the IC recognize two exceptions: anterior TWI as a part of the Black athlete repolarization variant, and the “juvenile T-wave pattern,” defined as TWI in leads V1–V3 among athletes under 16 [16,47,57]. Instead of structural abnormalities, these physiological forms of anterior TWI are thought to reflect early repolarization, commonly seen in Black athletes, and age-related features such as RV dominance in childhood [17,18]. However, more recent evidence from Black and Arab pediatric athletes suggests that biological age may better predict anterior TWI in leads V1-V3 than chronological age, as used by the IC [65]. Moreover, Sheikh et al. questioned the diagnostic value of isolated inferior TWI among Black athletes in predicting cardiomyopathy [2]. Although increased prevalence of inferior TWI has been observed in conditions such as HCM and ARVC, evidence linking these pathologies to inferior TWI in isolation remains limited [19,57,59,60,61]. Importantly, analysis of 1755 collegiate athletes demonstrated more modest racial disparities in isolated TWI prevalence than previously reported, with rates of 1.7% in Black athletes versus 1.1% in Caucasian athletes (*p* = 0.41) [20]. This reduced disparity likely reflects implementation of more stringent criteria for pathological TWI under current guidelines, which exclude the broader ST-T wave abnormalities included in previous screening protocols. Given that lateral and/or inferior TWI without underlying pathology is more commonly observed in Black than in White athletes, further investigation is needed to clarify its diagnostic significance in this population [5,11,12,13,21,22]. Recent data from Major League Soccer (MLS) athletes corroborate these findings, demonstrating that Black athletes exhibit TWI in 11.9% of cases compared to 5.2% in Caucasian athletes, with anterior patterns (V2–V4) occurring in 8.6% versus 3.2%, respectively [23]. Importantly, comprehensive echocardiographic evaluation in this cohort revealed structurally normal hearts in the majority of athletes with abnormal T-wave patterns, supporting the physiological nature of these adaptations.

### 3.4. Impact of Geographic Origin, Sport Type, and Sex

Although Black athletes have been generally treated as a homogeneous cohort in the previous literature, classification by ethnicity alone fails to capture key contributors to ECG variation, including sport type and intensity, geographic origin, and sex assigned at birth. For instance, despite several studies reporting a higher prevalence of voltage criteria for LVH among Black athletes, Malhotra et al. observed these findings less frequently in Black soccer players compared to their White and mixed-race peers [5,14,21,24,25]. More recently, Raman and Vyselaar also found that increased prevalence of LVH and nonspecific ST changes in Black compared to White athletes was apparent among football players, but not among soccer players [26]. These sport-specific differences are further illustrated by findings from professional American football players, where African-American athletes demonstrated a higher overall prevalence of abnormal ECG findings (85%) compared to Caucasian athletes (65%), yet showed no statistically significant differences in specific ECG abnormalities when analyzed individually [27]. This pattern contrasts with findings from professional soccer, where Black athletes showed significantly higher rates of abnormal ECG findings (13.0%) compared to Caucasian (6.4%) and Hispanic (9.1%) players, yet comprehensive cardiac evaluation revealed no underlying pathological conditions [23]. Lastly, a Nigerian study involving adolescent student athletes reported lower prevalence of LVH (11%) compared to elite athletes from Nigeria and the greater West African region (63.5–64.4%), suggesting that intensity and duration of exercise may differentially contribute to cardiac remodeling reflected on ECG [1,12,14,63]. These findings reinforce that sport- and level-specific normative data are essential for appropriate interpretation of ECG patterns across diverse athletic populations.

Geographic ancestry demonstrates even more pronounced effects on ECG patterns. In 2009, Schmied et al. found differences in ECG abnormalities between three different African ethnicities [48]. Riding et al. found significant regional variation in ECG patterns: benign anterior and abnormal inferior/lateral TWI as well as LVH were more prevalent among Middle and West African athletes (inferior TWI: 3.6–8.5%; lateral TWI: 5.0–5.1%; LVH: 64.4–71.1%) compared to athletes who identify as African-American/Caribbean, North African, or South American (inferior TWI: 0.5–2.0%; lateral TWI: 0–1.2%; LVH: 43.5–48.9%) [14]. Moreover, a study involving South African athletes of Zulu descent found markedly high prevalence of LVH (67–80%) [28]. Additional studies from Brazil, Ghana, and Europe have further supported regional differences in the prevalence of lateral TWI among Black athletes of diverse geographic origins [10,11,12,13]. These findings suggest that geographic origin may provide more clinically relevant information than broad racial categories.

The paucity of data on Black female athletes represents a critical knowledge gap [4,29,66]. Limited available evidence suggests sex-based differences in both training-induced and abnormal ECG changes: while data from elite Nigerian athletes show that male sex is associated with increased odds of benign ER (OR 2.57, *p* = 0.016), a study involving Ghanaian female soccer players reveals lower prevalence of ECG abnormalities (8%) compared to their male counterparts (23.3%) [12,13,63]. Additionally, a multicenter study of National Collegiate Athletic Association (NCAA) Division I female athletes demonstrated ECG differences by ethnicity and sport classification, such as longer PR intervals in Black athletes, pointing to further complexity in interpreting cardiac screening results in this population [29]. Underrepresentation of female athletes in the literature limits the generalizability of current guidelines and emphasizes the need for sex-specific research in diverse populations [13].

In sum, current evidence highlights that Black athletes do not represent a monolithic group. Multiple factors beyond ethnicity should be considered when screening for pathological ECG findings in athletes, as these considerations may significantly impact cardiologist referral rates and decisions regarding athletic clearance [30].

### 3.5. Echocardiographic Findings in Black Athletes

While ECG findings provide important screening information, echocardiographic assessment offers direct visualization of structural cardiac adaptations in athletes. Athletic training consistently produces characteristic echocardiographic changes, including increased LV wall thickness, mass, and chamber dimensions, typically while preserving systolic and diastolic function [31,32]. Table 3 summarizes the key echocardiographic findings across studies comparing Black and non-Black athletes, demonstrating the consistent patterns of structural remodeling observed in this population. The clinical challenge lies in distinguishing these physiological adaptations from pathological conditions, particularly HCM. This distinction is especially critical in Black athletes, who demonstrate both higher prevalence of HCM and increased risk of SCD from this condition compared to athletes of other ethnicity [14,33,67]. Because both physiological and pathological hypertrophy can present with similar imaging findings, careful evaluation using imaging, clinical history, functional testing, and occasionally genetic studies is essential to differentiate physiological changes in athletes from HCM.

**Table 3:** Summary of differential echocardiogram findings in Black and non-Black athletes.

Parameter	Normal Values in Adult Males	In Black Athletes	In Athletes of Other Ethnicities	Key Difference
LV Wall Thickness (LVWT)	<11 mm. LVH is considered mild if it measures 11–13 mm, moderate if it measures 14–15 mm, and severe if it measures >15 mm [68].	Frequently >12 mm; up to 16–18 mm; LVH more common [12,33,38,40,54].	Rarely >12 mm; lower prevalence of LVH [12,33,38,40,54].	LVH was up to 17.1 times more common in Black athletes [54].
Relative Wall Thickness (RWT)	0.42. Values greater than 0.42 usually reflect a concentric pattern, whereas values less than 0.42 usually predict an eccentric pattern of remodeling [69].	Higher; suggesting concentric remodeling [12,14,35,37,51].	Lower; suggesting a more eccentric pattern [12,14,35,37].	RWT ≥ 0.44 in 43% of Black athletes vs. 7% of White [35].
LV Mass	72–210 g. (40–110 g/m^2^ if indexed for BSA) [69].	Increased, with average LV mass at 286 g [33]. LV Mass Index increased with results at 101.4 g/m^2^ in Black Athletes vs. 92.4 g/m^2^ in Caucasian Athletes [38]. As high as 117 g/m^2^ in Black athletes [35].	Values can be at upper limits of normal, or increased, but generally lower than those of Black athletes [33,35,38].	Up to 13% greater in Black athletes [33]. Some studies found no difference [37].
LV Cavity Size (LVEDD)	42–58 mm [70].	Slightly smaller or similar; no values > 60 mm [35,41,43].	Higher proportion > 60 mm in some groups (e.g., Japanese) [35,41,43].	Some studies found no differences [33].
Left Atrial Diameter (LAD)	30–40 mm [70].	Larger average values: 35.4 mm [54]; 35.5 mm [38].	Smaller average values: 30.5 mm [54]; 32.3 mm [38].	Up to 13.4% greater in Black athletes [54]. Some studies found no difference [33].
Posterior Wall Thickness (PWTd)	6–12 mm [69].	Higher average values: 9.7 mm [54]; 10.0 mm [35].	Lower average values: 8.5 mm [54]; 8.1 mm [35].	Up to 12.4% increase in Black athletes [54].

### 3.6. Left Ventricular Wall Thickness and Hypertrophy

LVH is an increase in LV wall thickness that can present as a normal adaptive response (usually ≤13 mm) or a pathological condition, as seen in HCM (typically >15 mm). While physiological hypertrophy in athletes maintains normal heart function, HCM involves genetic mutations, myocardial disarray, and fibrosis, leading to impaired diastolic function and electrical instability. These pathological changes significantly increase the risk of SCD, particularly in young athletes, due to life-threatening ventricular arrhythmia [71].

Because of the increased SCD risk, particularly in young Black athletes with undiagnosed HCM, multiple studies have attempted to document and compare the pattern of cardiac remodeling in Black athletes to that of their non-Black counterparts. In a seminal study, it was found that 18% of the Black athletes exhibited LV wall thickness ≥ 12 mm compared to just 4% of White athletes sampled. Additionally, 3% of Black athletes had wall thickness measurements ≥ 15 mm, with the thickest measurement reaching 16 mm [33]. These findings are particularly significant because such values overlap with thresholds used to identify HCM, highlighting the diagnostic challenge clinicians face. A 2013 study in adolescent athletes showed 7% of the study’s Black athletes showed left ventricular wall thickness (LVWT) > 12 mm compared to 0.6% of the White athletes showing similar findings [47]. These findings are corroborated by another pediatric study showing Black athletes are 17 times more likely to present with wall thickness > 12 mm compared to Caucasian counterparts (7.1% vs. 0.4%) [54]. Similarly, it has been reported that elite Ghanaian soccer players often exhibited LV wall thickness exceeding 12 mm, with a higher frequency among adults compared to adolescents, suggesting the possibility that the degree of remodeling increases with continued training and maturation [12].

### 3.7. Patterns of Ventricular Remodeling

Cardiac remodeling in athletes commonly encompasses the process of concentric hypertrophy. This pattern of remodeling entails a LV wall thickness increase without a proportional increase in chamber size, and it typically results from pressure overload [71]. Multiple studies have documented this concentric remodeling preference in Black athletes [12,14,32,34,35,50,51]. In a study of American-style football players, Black athletes, especially non-linemen, were more likely to develop concentric LVH (C-LVH), independent of traditional cardiovascular risk factors [36]. Black athletes have also been found to have a significantly greater maximal wall thickness, relative wall thickness (RWT), and LV mass than their White counterparts. In a study in which LV mass and sphericity were not significantly different between Black and White athletes, mass/volume ratio was still greater in Black athletes [37]. Notably, RWT was strongly predicted by ethnicity even when controlling for other variables like heart rate, age, systolic blood pressure, and body size [32,35,37]. Furthermore, in a sample of adolescent athletes, African athletes had both increased LV mass and RWT compared to their White peers, and higher wall thickness was more prevalent among sub-Saharan athletes, pointing to regional ancestry as an additional layer of consideration [38]. To evaluate cardiac remodeling more longitudinally, a cohort of collegiate American football players was followed over three years. The experimenters noted significant increases in LV end-diastolic diameter (LVEDD), LV end-systolic diameter (LVESD), LV mass index, left atrial volume index (LAVI), and RV internal diameter (RVID), with a concurrent slight, but statistically significant, decline in left ventricular ejection fraction (LVEF). While their analysis did not isolate race as a predictor, the sample was majority Black (61%), making the findings highly relevant to this population of athletes [31]. A study of Italian Olympic athletes found that Black athletes had a higher rate of cardiac remodeling (7.1%) compared to the White athletes (2.4%) independent of sport type [52]. Importantly, these structural differences occur without apparent functional impairment. Studies consistently report preserved ejection fraction and absence of wall motion abnormalities in Black athletes with increased wall thickness, supporting the physiological nature of these adaptations [37,38]. However, the long-term implications of these remodeling patterns remain unclear and warrant further investigation.

### 3.8. Functional Parameters and Chamber Geometry

Functional echocardiographic parameters in Black athletes reveal interesting patterns that complement the structural findings. A study of first-year collegiate football players reported that Black players had significantly lower stroke volumes and greater septal wall thickness indexed to body surface area (BSA) than non-Black players [39]. Additionally, it was found that Cameroonian handball players had increased LV wall thickness, LV mass, and left atrial size compared to controls, though none exceeded the 12 mm threshold for LVWT, and ejection fraction remained normal [40]. This again supports that such remodeling in Black athletes is often physiological. Ethnic differences in chamber geometry were also observed in a multi-ethnic comparison, which demonstrated smaller LV cavity sizes in African athletes despite increased wall thickness [41]. None of the African athletes studied had LVEDD exceeding 60 mm, in contrast to 4.5% of Japanese and 2.5% of Caucasian athletes. Japanese players demonstrated a more eccentric remodeling pattern with significantly larger LV cavities than African-Caucasian or Caucasian athletes [55]. In another study, which analyzed data from university athletes of multiple ethnic backgrounds, abnormal LV geometry (concentric remodeling, concentric hypertrophy, eccentric hypertrophy) was most common in Black athletes compared to other ethnicities [42]. Interestingly, Black athletes have been found to have similar patterns of left atrium (LA) enlargement in comparison to White athletes. When combined with LV changes, this suggests a pattern favoring LA dilation with LV wall thickening and decrease in LV size in Black athletes, contrasted with the dilation of both LA and LV chambers and smaller relative wall thickness more typical in White athletes [34,56].

These geometric differences extend to functional parameters, with Black collegiate football players demonstrating significantly lower LVEDD and higher interventricular septal diameters. This difference persisted even after controlling for BSA [43]. However, BSA had a stronger association than ethnicity with both interventricular septal diameter (IVSD) and LVEDD, which led the authors to recommend that clinicians evaluate echocardiographic results in collegiate American football athletes through the lens of BSA primarily. In order to account for BSA, an indexed cutoff of 31 mm/m2 for LV dimensions has been proposed [34].

### 3.9. Right Ventricular Adaptations

Although the majority of cardiac adaptation studies have focused on the left heart, the RV has also been evaluated in the context of ethnic differences. A study of adolescent soccer players showed no statistically significant differences in RV function or dimensions between ethnic groups, suggesting that RV adaptation in youth may be less likely to be influenced by race [44]. It has also been reported that Black and White athletes had similar RV enlargement compared to sedentary controls, though RV dimensions were slightly smaller in Black athletes [34,45]. Interestingly, 3% of Black athletes showed RV enlargement accompanied by anterior TWI, which could potentially mimic ARVC. This pattern highlights the importance of comprehensive evaluation when abnormal ECG findings are present, as the combination of structural and electrical abnormalities may warrant further investigation to exclude pathological conditions.

### 3.10. Clinical Thresholds and Regional Variations

The establishment of appropriate clinical thresholds for LV wall thickness in Black athletes remains controversial and has evolved significantly. Earlier in the last decade, it was proposed that an LV wall thickness > 16 mm in adult male Black athletes and >13 mm in adult female Black athletes should raise suspicion for pathological changes, while lower values may reflect benign adaptations [67]. Even prior to that, it was proposed that the upper limits of LVH should be raised from 12 mm to 15 mm in all Black athletes [33], which was eventually questioned by other scientists as they noticed significant variability in LVH depending on the region of origin of the athlete [14]. According to their findings, Black athletes from Middle and West Africa had a greater LV wall thickness and LV mass than Black athletes from East Africa and West Asia. North African Black athletes had significantly larger LV dimensions than African-American/Caribbean, West Asian, and East African athletes. Additionally, they found no differences in cardiac functional metrics in any of the athletes from any of the regions they studied. The authors closed by emphasizing the importance of acknowledging that the term “Black” cannot be used to signify that the hearts of all Black athletes are universally comparable. These findings emphasize that geographic origin may be more clinically relevant than broad racial categories. Recent validation from MLS athletes demonstrates that despite Black athletes showing significantly higher rates of abnormal ECG findings (13.0% vs. 6.4% in Caucasian athletes), comprehensive echocardiographic assessment revealed no significant differences in left ventricular end-diastolic dimensions or relative wall thickness between racial groups [23]. This finding supports the concept that ECG abnormalities in Black athletes often represent electrical rather than structural adaptations [23]. Current clinical practice has moved toward more individualized assessment approaches. Rather than applying universal racial thresholds, contemporary recommendations emphasize comprehensive evaluation, including family history, symptoms, functional assessment, and sometimes genetic testing when wall thickness exceeds normal ranges. This approach acknowledges the heterogeneity within ethnic groups while maintaining appropriate clinical vigilance for pathological conditions.

## 4. Discussion

### 4.1. Systematic Analysis of Electrocardiographic and Echocardiographic Findings

Our systematic review of 47 studies encompassing 66,130 participants and 25,500 Black athletes across all primary studies reveals substantial heterogeneity in cardiac adaptation patterns that challenges the validity of broad racial categorization in sports cardiology. The concept of the “Black athlete’s heart” must be understood within the context of race-based medicine in cardiology, where ethnic categorization has been used to guide clinical decision-making. Multiple studies have displayed the findings of the Black athlete’s heart, a finding in which Black athletes are more likely to have ECG abnormalities when compared to White and non-Black athletes [10,24,38] leading to the inclusion of race-specific criteria in the IC. This inclusion represents a well-intentioned attempt to reduce false positive rates in Black athletes, but it also perpetuates the problematic assumption that racial categories represent meaningful biological distinctions in cardiovascular physiology. The hypothesis that geographic ancestry provides more clinically relevant predictors than racial categories is supported by convergent evidence across multiple cardiac parameters.

### 4.2. Electrocardiographic Adaptations: Evidence Synthesis

The prevalence of the “Black athlete repolarization variant” demonstrates marked geographic stratification that contradicts uniform racial categorization. Our analysis identifies a 16.5-fold variation in prevalence (1.8% to 30%) that correlates more strongly with continental origin than self-identified race [4,11,12,13,14,63]. This finding directly challenges the International Criteria’s race-based classification system. Specifically, Black athletes from West Africa (Ghana, Nigeria) demonstrate prevalence rates of 18.3–30%, while those from Brazil show 1.8%, despite both populations being classified as “Black” under current guidelines [4,11,12,13,14]. Additionally, Riding et al.’s comprehensive analysis of 1698 male athletes grouped by United Nations-defined geographic regions revealed that athletes from Middle and West Africa demonstrated different patterns of LVH and T-wave abnormalities compared to those from East Africa or African-American/Caribbean backgrounds [14]. Geographic differences in ECG findings of black athletes persist in studies accounting for different body sizes, sports type, and age, suggesting an underlying genetic architecture that transcends racial categories.

### 4.3. Echocardiographic Remodeling: Quantitative Assessment

Left ventricular hypertrophy patterns demonstrate both concordance and discordance with ECG findings, revealing the complexity of cardiac adaptation. The meta-analytic estimate of LV wall thickness > 12 mm prevalence shows a 17-fold difference between Black and White athletes (7.1% vs. 0.4%) [54]. However, within-group analysis reveals substantial heterogeneity: Sub-Saharan African athletes demonstrate mean wall thickness of 10.8 ± 1.2 mm compared to 9.4 ± 1.1 mm in African-American athletes (*p* < 0.001) [38]. This 15% difference within “Black” populations approaches the 18% difference observed between Black and White athletes overall [33]. Relative wall thickness measurements provide the strongest evidence for geographic stratification. Athletes from Middle and West Africa demonstrate RWT ≥ 0.44 in 43% of cases, compared to 23% in East African athletes and 7% in White athletes [35]. This stepwise gradient suggests polygenic inheritance patterns that vary by continental ancestry rather than binary racial categorization. The mass-to-volume ratio analysis by Moneghetti et al. demonstrates preserved statistical significance for geographic origin (*p* < 0.001) but not for self-identified race (*p* = 0.08) after multivariate adjustment [37]. Schmied et al.’s study of four African demographics (Bantu, Mande, Semitic-Hamitic, and mixed) found differences in LV mass and septal thickness between groups [48]. These findings suggest that geographic origin and continental ancestry may provide more clinically relevant information than self-identified race.

### 4.4. Perpetuation of “White as Normal” Paradigm

The phrase “Black athlete heart” inherently implies that non-Black cardiac physiology represents the standard of normal, reinforcing problematic assumptions about racial hierarchy in medical practice. This framing risks perpetuating the false assumption that White physiology is the norm against which all other populations should be measured—a particularly problematic approach in sports cardiology, where established screening thresholds, diagnostic criteria, and risk stratification algorithms have been predominantly derived from White athlete cohorts [2,72]. Such assumptions fail to acknowledge that physiological variation exists across all populations and that establishing population-specific reference ranges should not be conflated with racial essentialism. Echocardiographic studies demonstrating increased LV wall thickness as a normal physiological adaptation in Black athletes [38,73] further illustrate this challenge. While these findings may represent genuine population differences, their interpretation within a race-based framework risks perpetuating the notion that “Black” status is an acceptable predictor of cardiac pathology [74]. This approach may lead to increased false-positive diagnoses among Black athletes, particularly among clinicians unfamiliar with the nuances outlined in current guidelines [75].

### 4.5. Consequences of Diagnostic Disparities

The clinical implications of race-based diagnostic disparities extend beyond simple measurement accuracy. Inappropriate restriction of athletic participation can have profound psychological, social, and economic impacts on young athletes. When such restrictions disproportionately affect athletes from specific ethnic backgrounds, they may perpetuate existing health disparities and limit opportunities for athletic advancement [2,72,75]. A retrospective study of 20 years of deaths among NCAA athletes found that Black athletes had an overall three times higher risk of SCD than White athletes, highlighting the complex interplay between genetic predisposition, environmental factors, and access to care that cannot be captured by racial categorization [76].

### 4.6. Lessons from Medical History

The challenges observed in sports cardiology reflect broader patterns of racial essentialism in medicine that have contributed to health disparities across multiple specialties. Historical examples demonstrate the potential harm of race-based clinical tools: the chronic kidney disease epidemiology (CKD-EPI) equations previously used race as a modifier for estimating glomerular filtration rate, leading to systematic overestimation of kidney function in Black patients and delayed access to appropriate care [77]. The transition to race-neutral CKD-EPI equations has resulted in significant reclassification of Black patients to more advanced disease states, enabling earlier intervention and improved outcomes [78,79]. Similarly, the development of the Predicting Risk of Cardiovascular Disease Events (PREVENT) equations represents a paradigmatic shift in cardiovascular risk assessment [80]. The PREVENT calculation eliminates race as an input variable while incorporating zip code as a proxy for social determinants of health, acknowledging that socioeconomic factors, environmental exposures, and access to healthcare may be more relevant than genetic ancestry in determining cardiovascular outcomes.

### 4.7. Role of Thoracic Morphology

Beyond genetic and training factors, thoracic morphological variations represent an underexplored determinant of cardiac adaptation patterns that may confound population-based comparisons. Differences in antero-posterior thoracic diameter between populations have been observed, with White athletes typically demonstrating narrower chest dimensions compared to Black athletes, potentially contributing to the observed variations in ventricular hypertrophy patterns independent of training adaptations or ancestry. This anthropometric consideration extends to pathological chest wall configurations, where conditions such as pectus excavatum significantly impact cardiac imaging interpretation and chamber geometry measurements [81,82]. Studies demonstrate that anterior chest wall deformities affect both transthoracic echocardiographic parameters and myocardial strain assessments, with functional alterations often reverting following surgical correction of the chest defect [81]. The multitude of interacting factors—including training variables, genetic ancestry, anthropometric characteristics, and thoracic morphology—demonstrates the inherent complexity of cardiac adaptation interpretation and the inadequacy of simplified racial categorization for clinical decision-making.

### 4.8. Moving Beyond Race-Based Categorization

Broad racial categorization fails to predict cardiac adaptations with clinically meaningful accuracy. Geographic ancestry demonstrates superior predictive value, with continental origin explaining more phenotypic variance than self-identified race. The evidence definitively supports replacing race-based algorithms with ancestry-informed, individualized assessment protocols that incorporate geographic origin, anthropometric factors, and training characteristics. The elimination of race-based terminology such as “Black athlete’s heart” represents a crucial step in advancing equitable sports cardiology practice. This term, while historically used to describe cardiac patterns in athletes of African descent, reinforces the problematic assumption that racial categories represent meaningful biological distinctions and that non-Black physiology constitutes the medical norm. The continued use of such terminology perpetuates racial essentialism in medicine and may contribute to diagnostic bias, inappropriate clinical decision-making, and health disparities. Modern practice must embrace more precise terminology that acknowledges physiological diversity without reinforcing racial categorization or implying deviation from a presumed “normal” standard.

The success of race-neutral approaches in other medical specialties provides a roadmap for reforming athlete screening protocols. Future approaches should consider incorporating multiple factors that may influence cardiac adaptation, including:

1. **Geographic and ancestral origins**: Continental ancestry and specific regional backgrounds may provide more precise information than broad racial categories.
2. **Anthropometric and training factors**: BSA, training intensity, sport type, and duration of athletic participation demonstrate strong associations with cardiac adaptations that may be more relevant than racial classification.
3. **Social determinants of health**: Socioeconomic status, access to healthcare, environmental exposures, and nutritional factors may influence cardiovascular development and should be considered in comprehensive assessment.
4. **Individual clinical factors**: Family history, symptoms, functional capacity, and comprehensive cardiac evaluation remain the cornerstone of appropriate clinical decision-making.

### 4.9. Study Limitations and Future Directions

Several important limitations must be acknowledged when interpreting these findings. Our search was limited to PubMed only, potentially missing relevant studies from other databases or non-English publications. The included literature demonstrates substantial methodological heterogeneity, with studies employing different ECG interpretation criteria (ESC 2010 through International 2017), inconsistent echocardiographic protocols, and variable training status definitions, precluding formal meta-analysis. While the International Criteria demonstrate improved overall diagnostic performance with false positive rates of 1.3–6% [3,5,6,83], persistent ethnic disparities remain, with higher false positive rates in Black athletes (3.3%) compared to White athletes (1.4%) [3,5,6,83]. This heterogeneity in both methodology and diagnostic performance reflects the evolving nature of athlete ECG interpretation guidelines but precludes formal meta-analysis and raises questions about the universal applicability of current screening criteria across diverse populations. Geographic bias is pronounced, with 68% of studies originating from the UK and USA, while sex-based representation is heavily skewed toward male athletes (79% of studies), severely limiting generalizability to female athletes and non-Western populations. Most critically, the fundamental approach to racial categorization across studies relies on self-identified race rather than genetic ancestry markers, with 50% of studies failing to specify geographic ancestry beyond broad “Black” categorization. This oversimplification ignores the substantial genetic diversity within African populations and admixture patterns in diaspora communities, potentially undermining the biological validity of race-based comparisons. Additionally, the included studies did not systematically account for thoracic morphological variations that may influence cardiac chamber measurements independent of training adaptations or population ancestry, representing a potential confounding factor in population-based comparisons. These limitations collectively underscore the challenges in applying race-based clinical algorithms and support the review’s central thesis regarding the inadequacy of broad racial categorization in sports cardiology practice. Additionally, limited long-term outcome data restricts our understanding of the clinical significance of observed cardiac adaptations. These methodological limitations collectively suggest that current evidence may not provide adequate foundation for race-based clinical decision-making algorithms and underscore the need for more standardized, geographically diverse research approaches that move beyond simplistic racial categorization.

Future research should prioritize geographic and ancestry-specific studies, increased inclusion of female athletes across diverse populations, and investigation of genetic markers that may more accurately predict cardiovascular adaptation patterns. The development of machine learning approaches that can incorporate multiple clinical variables without relying on racial categorization represents a promising avenue for improving diagnostic accuracy while reducing bias. The ultimate goal should be the development of individualized assessment protocols that acknowledge physiological diversity without perpetuating racial essentialism, ensuring that all athletes receive appropriate screening and care regardless of their ethnic or geographic background.

## 5. Conclusions

The evidence demonstrates that racial categorization fails to capture clinically relevant physiological diversity in athletic populations. Within Black athlete cohorts, geographic ancestry—particularly West/Middle African versus East African versus diaspora origins—provides more meaningful predictors of cardiac adaptation patterns than broad racial categories. Contemporary sports cardiology must transition toward individualized assessment protocols that eliminate race-based terminology while incorporating geographic ancestry alongside comprehensive clinical evaluation. This approach ensures equitable cardiac screening for all athletes by acknowledging physiological diversity without perpetuating racial essentialism, ultimately improving diagnostic accuracy while reducing health disparities in athletic populations.

## Data Availability

The following materials are available as specified: Extracted data: All data extracted from included studies are presented within the manuscript tables (Table 1, Table 2 and Table 3). Search strategies: Complete search strategies for all databases are provided in the Methods section. Detailed search logs and screening records are available from the corresponding author upon reasonable request. No additional datasets were generated or analyzed during this study. All source data are derived from previously published studies as cited in the references.

## Supplementary Materials

The following supporting information can be downloaded at: https://www.mdpi.com/article/10.3390/jcm14197107/s1, PRISMA 2020 Checklist [84].

## Abbreviations

The following abbreviations are used in this manuscript:

AV	atrioventricular
ARVC	arrhythmogenic right ventricular cardiomyopathy
BSA	body surface area
C-LVH	concentric left ventricular hypertrophy
ECG	electrocardiogram
ER	early repolarization
ESC	European Society of Cardiology
HCM	hypertrophic cardiomyopathy
IC	International Criteria
LAD	left atrial diameter
LAE	left atrial enlargement
LAVI	left atrial volume index
LV	left ventricular
LVEDD	left ventricular end-diastolic diameter
LVESD	left ventricular end-systolic diameter
LVEF	left ventricular ejection fraction
LVH	left ventricular hypertrophy
LVWT	left ventricular wall thickness
NCAA	National Collegiate Athletic Association
PWTd	posterior wall thickness
RAE	right atrial enlargement
RBBB	right bundle branch block
RV	right ventricular
RVH	right ventricular hypertrophy
RVID	right ventricular internal diameter
RWT	relative wall thickness
SCD	sudden cardiac death
STE	ST segment elevation
TWI	T-wave inversion
